# A Deeper Curse: A Hirschsprung Patient's Evaluation Unmasks a Rare Association with Congenital Central Hypoventilation Syndrome and Neuroblastoma

**DOI:** 10.1055/s-0042-1758826

**Published:** 2022-11-29

**Authors:** Shimon Eric Jacobs, Laura Tiusaba, Elizaveta Bokova, Tamador Al-Shamaileh, Teresa Lynn Russell, Emily C. Rutan, Harutyun Haroyan, Yong Wang, Christina Feng, Andrea Badillo, Marc A. Levitt

**Affiliations:** 1 Division of Colorectal and Pelvic Reconstructive Surgery, Children's National Hospital, Washington, District of Columbia, United States; 2 Department of General Surgery, King Hussein Cancer Center, Amman, Jordan; 3 Department of Radiology, Children's National Hospital, Washington, District of Columbia, United States; 4 Department of Pediatric Surgery, Xinhua Hospital Affiliated to Shanghai Jiaotong University School of Medicine, Shanghai, China

**Keywords:** congenital central hypoventilation syndrome, Hirschsprung disease, neuroblastoma, Haddad syndrome, PHOX2B gene

## Abstract

We present a rare case of a 2-year-old male patient referred for primary evaluation of constipation and ultimately treatment of Hirschsprung disease (HSCR) whose preoperative workup incidentally revealed a posterior paraspinal mass. Following the biopsy of the mass, the patient exhibited hypoventilation and hypoxia requiring a delayed extubation, raising suspicion for congenital central hypoventilation syndrome (CCHS). We focus on the known history of associations between HSCR and CCHS, in addition to recently found genetic mutations in paired-like homeobox 2B that link HSCR, CCHS, and neuroblastoma.

## Introduction

Hirschsprung disease (HSCR) is characterized by intestinal aganglionosis, most commonly of the large intestine, causing functional obstruction. The pathophysiology is thought to be related to a defect in cranial to caudal migration of neural crest cells resulting in a failure to colonize the affected regions. 1 2 Additionally, it is hypothesized that disruption of neural crest cell proliferation, migration, and differentiation into ganglion cells or destruction may be contributors to the disease. 3 In approximately 70% of cases, HSCR occurs in isolation, 12% with a chromosomal defect (mostly Trisomy 21), and in the remainder as part of a genetic syndrome. 4 Inheritance of HSCR is complex, often non-Mendelian, and with variable penetrance; however, through extensive research several single-gene mutations have been identified. These include genes important for migration, proliferation, and differentiation of neural crest cells, such as the RET proto-oncogene, endothelin receptor B, SRY-box transcription factor 10, and paired-like homeobox 2B (PHOX2B), among others. 5 6 The phenotypic expressions of these mutations are varied and can result in comorbid conditions in addition to HSCR. We present a case highlighting the link of HSCR with other neurocristopathies.

## Case Presentation

A 2-year-old full-term male was referred to our institution for surgical treatment of HSCR. His prior history was notable for a prolonged neonatal intensive care unit stay after birth, attributed to seizures due to cerebral hematomas, and a brief intubation period with subsequent supplemental oxygen support postextubation during his hospitalization. At the age 17 months, he developed abdominal distention and enterocolitis. Following enterocolitis treatment, he underwent a rectal biopsy at the referring institution, which suggested HSCR. The patient's obstructive symptoms were managed with laxatives, without the need for irrigations.

**Fig. 1:**
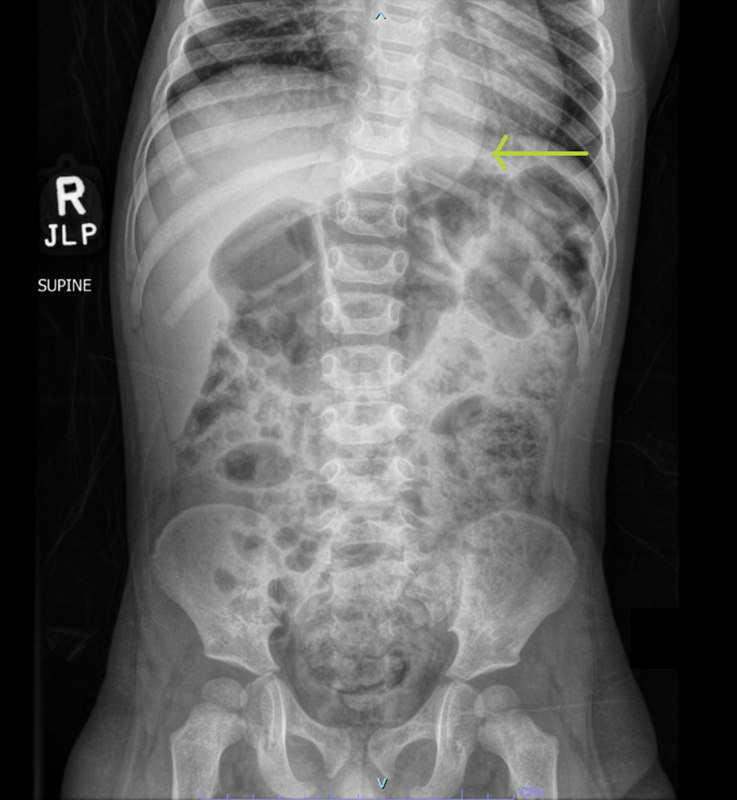
A supine abdominal X-ray upon presentation incidentally demonstrates a smooth, circular paraspinal mass at the level of the diaphragm (arrow).

**Fig. 2:**
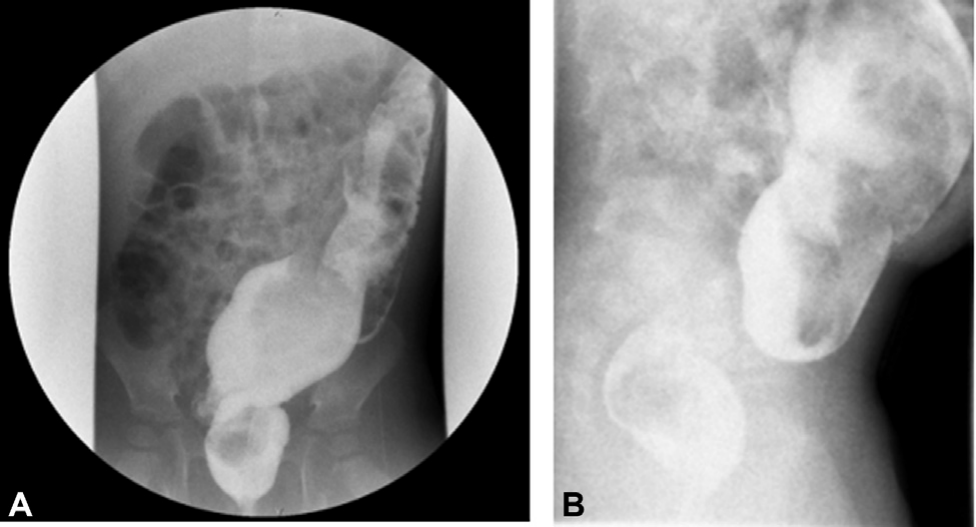
The ( **A** ) anteroposterior and ( **B** ) lateral views of the contrast enema demonstrate reversed rectosigmoid ratio—a dilated sigmoid colon relative to the rectum suggestive of a transition zone.

**Fig. 3:**
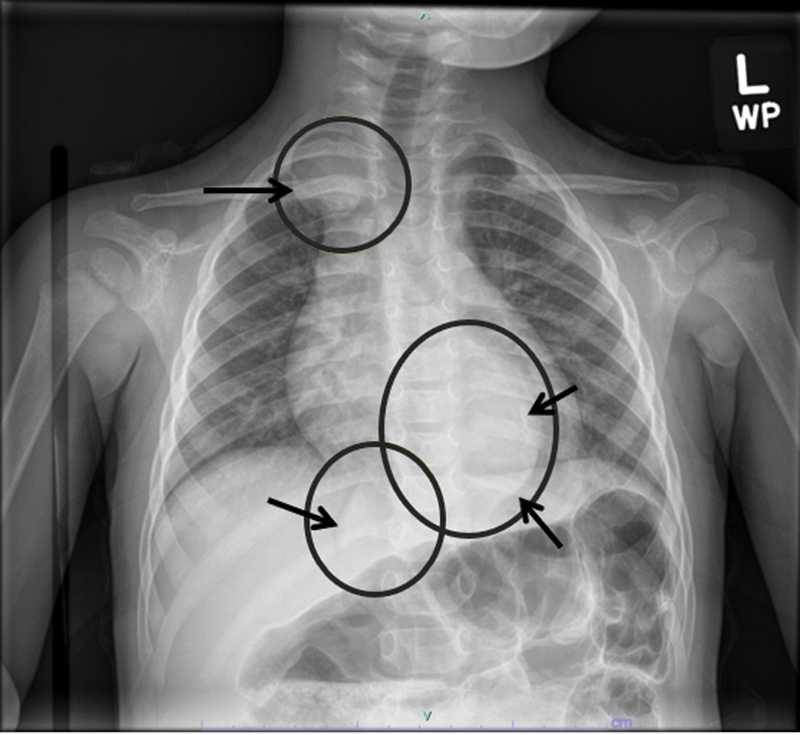
A dedicated chest X-ray showing three masses annotated with circles and arrows.

In our initial evaluation, a plain abdominal X-ray ( Fig. 1 ) and contrast enema ( Fig. 2 ) were performed to assess the stool burden, a transition zone, and any bowel dilation. The abdominal film incidentally showed a rounded left paraspinal mass at the level of the diaphragm, prompting us to obtain a formal chest X-ray ( Fig. 3 ). Three thoracic masses were identified, confirmed with a chest computed tomography, raising concern for a multifocal neoplastic process, such as neuroblastoma.

The patient subsequently underwent an image-guided biopsy of the dominant paraspinal mass along with a repeat rectal biopsy to confirm HSCR, all performed under general anesthesia. Upon emergence from anesthesia, the patient had poor respiratory effort and hypoxia requiring reintubation, thought initially to be related to oversedation or bronchospasm. After eventual successful extubation approximately an hour later, the patient was monitored in the postanesthesia care unit overnight. The hospitalist consulted raised a concern for congenital central hypoventilation syndrome (CCHS) due to its known association with HSCR and neuroblastoma, prompting additional evaluation by a pulmonologist.

The paraspinal biopsy confirmed a neuroblastoma, and rectal biopsy supported the diagnosis of HSCR. Multidisciplinary discussion among colorectal surgery, oncology, pulmonology, and anesthesia team members led to a treatment plan of transanal rectosigmoid pull-through prior to neuroblastoma treatment. A frozen section at 16cm from the anus lacked ganglion cells, an additional biopsy at 25cm showed ganglion cells and no nerve hypertrophy, and the final pull-through segment was 35cm. The patient was admitted to the pediatric intensive care unit following his pull-through procedure, intubated, with successful extubation on postoperative day 1 with medical pulmonary optimization. On the floor, the patient required nighttime oxygen supplementation, which was weaned off before discharge on postoperative day 4.

The patient proceeded to undergo further staging workup and treatment for the neuroblastoma, including metaiodobenzylguanidine scan, port placement, induction chemotherapy, and genetic testing. Tumor analysis revealed a poorly differentiated, low MKI (mitosis-karyorrhexis index) specimen consistent with unfavorable histology. The tumor showed nonamplification of MYCN (N-myc proto-oncogene protein) and a 11q loss of heterozygosity. Without image-defined risk factors, the patient was determined intermediate risk. He subsequently underwent four cycles of chemotherapy, achieving a partial response meeting criteria for cessation of therapy. Six months post-treatment, he remains without progression of disease and continues with surveillance imaging every 3 months.

After a period of hypermotility postoperatively, he was able to make dietary and medication changes to slow down to four to five bowel movements daily. In his initial treatment, loperamide was used as first-line antimotility agent; however, he had an episode of severe hypoventilation with mental status depression after its use requiring narcotic reversal and supportive oxygen therapy and was thus discontinued. A sleep study was performed given his prior hypoventilation and concern for CCHS, showing multiple central apneic and obstructive apneic events, but without elevation in partial pressure of carbon dioxide level expected in CCHS. Genetic test was sent for expert review and was determined not to have any mutations in PHOX2B classic for CCHS.

## Discussion

CCHS is a sleep-related hypoventilation syndrome due to autonomic dysregulation of the respiratory drive, prolonged central apneas, and diminutive tidal volumes resulting in a mismatch between CO _2_ elimination and metabolic production leading to hypercapnia and often hypoxia. The disorder is also known as “Ondine's Curse,” alluding to the fate of a human male who loved but betrayed a mythological water nymph. In one version of the tale, the man was cursed to die if he was to fall asleep, but the description of dysautonomia in CCHS is well-captured in lines of the play “Ondine” by Giraudoux: “Since you went away, I've had to force my body to do things it should do automatically. I no longer see unless I order my eyes to see (…) I have five senses, thirty muscles, even my bones to command; it's an exhausting stewardship. If I relax my vigilance for one moment, I may forget to hear or to breathe.” 7

Patients with CCHS commonly present in the newborn period with cyanosis, especially during sleep, without apparent distress or effort to compensate. This patient's prolonged neonatal intensive care unit stay and intubation were reported to be related to seizures and cerebral hematoma, but raised suspicion for undiagnosed CCHS during that period.

The association between CCHS and HSCR is known as Haddad syndrome, described first in 1978 in three cases. 8 All patients were ventilator-dependent until their eventual demise at 2 to 5 months of age, and had undergone pull-through or colostomy for their HSCR. Also noted at autopsy, although not explored further at the time, was the discovery in one of those patients of numerous ganglioneuroblastomas in the thoracic sympathetic chains and bilateral adrenal glands.

CCHS is a monogenic syndrome characterized by mutations of the PHOX2B gene at chromosome 4p12, inherited in an autosomal dominant manner when not sporadic. Genotyping of the mutation and classification into a polyalanine repeat mutation (PARM, 90% of cases) or non-polyalanine repeat mutation (NPARM, 10% of cases) is essential in the diagnosis and prognostication given more severe phenotypes in NPARMs. HSCR is seen in approximately 20 to 30% of CCHS cases, but greater than 80% of NPARM had HSCR compared to 10 to 20% in PARM genotypes. 9 10 Furthermore, tumors of neural crest origin are seen in 50% of NPARM compared to 1% of PARM genotypes. 10 Thus, genotyping and classification of the mutation dictate initial testing strategies and surveillance for the predicted phenotype.

The PHOX2B gene is essential in the development of most relays of the autonomic nervous system, including all autonomic neural crest derivatives, suggesting variable expression of a single genetic abnormality could be a common cause to these three neurocristopathies—PHOX2B is the main disease-causing gene for CCHS as described above, and the first gene for which germline mutations have been demonstrated to predispose to neuroblastoma, and HSCR was associated with PHOX2B gene single-nucleotide polymorphism. 11 12

Simultaneous occurrence of neuroblastoma, HSCR, and CCHS, referred to as NB-HSCR-CCHS cluster, is extremely rare. While at least 1000 cases of CCHS have been reported, the largest series found 6/188 (3.2%) patients with CCHS also had HSCR and a tumor of the sympathetic nervous system (such as neuroblastoma. 13 The majority of NB-HSCR-CCHS cluster cases occur in NPARM genotypes and the neuroblastoma is commonly multifocal, as in this patient. 13 14 In our case, we proceeded with the transanal pull-through before chemotherapy in order to avoid the risk of recurrent enterocolitis and the possible need for rectal irrigations in the setting of immunosuppression. Reports of a milder natural course of neuroblastoma despite multifocality have been made in patients with underlying PHOX2B with neurocristopathy syndromes. 15 According to the International Neuroblastoma Risk Group classification, our patient will be treated as an intermediate risk, given that his tumors have N-MYC nonamplification and localization to the thoracic cavity without image-defined risk factors.

After expert review of the genetic testing result for PHOX2B gene in this case showed that no PARM mutations were present, and coupled with sleep study results inconsistent with CCHS, no further genetic testing was deemed necessary. In the case of a diagnosis of CCHS, patients are recommended to undergo routine in-patient assessments of awake and sleep oxygenation and ventilation. 10 CCHS does not respond to pharmacologic stimulants, does not resolve spontaneously, and is not expected to improve with age, thus continual assessment of the individual's physiology is needed. Supportive measures such as positive pressure ventilation via tracheostomy, bi-level positive airway pressure, negative pressure ventilation, or diaphragmatic pacing may be necessary on an individualized basis. 10 Additional annual testing recommended includes neurocognitive testing, 72-hour Holter recording and echocardiography, and a contrast enema and possible rectal biopsy to rule out HSCR. 10 Imaging surveillance for neural crest tumors is guided by the underlying PHOX2B mutation. 10

## Conclusion

This case demonstrates a rare association of HSCR, multifocal neuroblastoma, and suspected CCHS. A multidisciplinary approach was essential to devise the optimal sequence of evaluation and planned treatment for all maladies.

## Notes

New Insights and the Importance for the Pediatric Surgeon Clinicians who treat patients with Hirschsprung disease should be aware of associated genetic and chromosomal syndromes—this case highlights an association with multifocal neuroblastoma and suspected congenital central hypoventilation syndrome. A multidisciplinary approach was essential to devise the optimal sequence of evaluation and planned treatment for all maladies.
